# A Simple Method for Fabricating Ink Chamber of Inkjet Printheads

**DOI:** 10.3390/mi13030455

**Published:** 2022-03-17

**Authors:** Zheguan Huang, Yang Tang, Zhibin Liu, Xiaofei Zhang, Yan Zhou, Yonglin Xie

**Affiliations:** 1 Key Laboratory of Nanodevices and Applications, Suzhou Institute of Nano-Tech and Nano-Bionics, Chinese Academy of Sciences, Suzhou 215123, China; zghuang09@163.com (Z.H.); xfzhang2013@sinano.ac.cn (X.Z.); yzhou2013@sinano.ac.cn (Y.Z.); 2 Gusu Laboratory of Materials, Suzhou 215123, China; ytang2018@sinano.ac.cn; 3 School of Nano-Tech and Nano-Bionics, University of Science and Technology of China, Hefei 230026, China; zbliu2019@sinano.ac.cn

**Keywords:** ink chamber, printhead, molding, film transfer, lithography

## Abstract

The process of fabricating chambers is becoming more important for inkjet printheads. However, there are some problems with the majority of present fabrication methods, such as nozzle structural deformation, blocked chambers, and collapsed chambers. In this paper, we propose a new process for preparing printhead chips by bonding tantalum nitride thin-film heaters and SU-8 chamber film using UV curing optical adhesive. This process simplifies the preparation process of printhead chips and overcomes the limitations of the traditional adhesive bonding process. Firstly, a chamber film was prepared by the molding lithography process based on a PDMS mold. The chamber film was then bonded with the membrane heater by the adhesive bonding process based on film transfer to form a thermal bubble printhead chip. Finally, the chip was integrated with other components to form a thermal inkjet printhead. The results show that the overflow width of bonding interface of 3.10 μm and bonding strength of 3.3 MPa were achieved. In addition, the printhead could stably eject polyvinyl pyrrolidone binder droplets, which are expected to be used for binder-jetting printing of powder such as ceramics, metals, and sand molds. These results might provide new clues to better understand the adhesive bonding process based on film transfer and the new applications of inkjet printheads.

## 1. Introduction

Inkjet printing is an attractive printing technology due to its several advantages such as high speed, quiet operation, and compatibility with a variety of substrates [1]. It has been used in many applications, such as sensor [2], solar cell [3], super capacitor [4,5], and electronic circuit [6]. Currently, inkjet printing comprises thermal inkjet (TIJ), piezoelectric inkjet (PIJ), and electrostatic, etc. Among them, the TIJ and PIJ devices are most nature and commonly used in inkjet printers. The TIJ printhead has a much lower cost, higher nozzle density, and higher print speed than the PIJ printhead [7]. At present, thermal bubble printheads have been used in graphene, ceramic, and metal. Huang et al. fabricated the reduced graphene circuits on Si/SiO_2_ substrate by using thermal bubble inkjet printing of water-based graphene oxide (GO) and graphene inks on heated substrate; the conductivity of the printed graphene pattern is 269 S/m [8]. Huang et al. also fabricated alumina ceramic and graphene circuits-on-ceramics by thermal bubble inkjet technology; the 3D-printed α-Al_2_O_3_ ceramic achieved a maximum density of 95.2%, volume shrinkage rate of 32%, and maximum compression strength and flexural strength of 89 MPa and 49.4 MPa, respectively [9]. Tang et al. used a thermal bubble printhead to fabricate Ti6Al4V alloy parts with excellent properties; the sintered sample with mixed powder at 1420 °C for 2 h achieved a maximum density of 95.2%, linear shrinkage of 13.4%, compression strength of 1021 MPa and yield stress of 589 MPa, and elongation of 4.15% [10]. In addition, thermal bubble inkprinting technology has been applied in cell bioprinting because of its unique mechanism [11,12]. Such technology has little damage to cells [13].

The core component in a TIJ printing system is the printhead, which includes the heater, ink chamber, inlet manifold, nozzle, and wire electrode [1]. The printhead is a successful product of micromachining technology. At present, micro-electro-mechanical systems (MEMS) technology has become the mainstream method of inkjet printhead manufacturing [14]. It is based on photolithography, deposition, epitaxy, doping, etching, packaging, bonding, and other two-dimensional or three-dimensional processing methods as the basic process steps to manufacture complex three-dimensional devices [15,16]. The process of fabricating chambers is becoming more important for the inkjet printhead. However, there are some problems with the majority of present fabrication methods, such as nozzle structural deformation, blocked chambers, and collapsed chambers [17,18,19]. As a semi-closed three-dimensional structure, the printhead chamber is one of the difficulties in the whole preparation process of an inkjet head. Currently, the common preparation technology of the inkjet printhead chamber includes a sacrificial layer, thermal bonding, and adhesive bonding technique [19,20,21].

In the past decades, some research on the preparation technology of inkjet printhead chambers has been reported. Liou et al. (2010) fabricated a monolithic thermal inkjet printhead based on MEMS fabrication processes including the sacrificial layer process, and the results showed that the major advantage of the inkjet chip assembly processes is improved throughput [22]. Wang et al. (2019) fabricated chambers of inkjet printheads by thermal bonding an SU-8 nozzle plate with a suitable crosslinked degree; the results showed that the optimized bonding strength of 0.57 MPa and chamber deformation rate of 4.35% were obtained in the final inkjet printhead [17]. However, the sacrificial layer technique suffers from some problems, including a long fabrication process and complicated workmanship due to the difficult removal of the sacrificial layer caused by the semiclosed chamber structure and the need for graphical layers of sacrifice and structure [23]. The thermal bonding technique requires a high temperature and high pressure in the bonding process, leading to deformation and damage of the structure [24]. Although the adhesive bonding approach has some disadvantages, such as the need to design the joints in the proper way or to have adherent substrates, the requirement for specific clamping devices to fix the joint during cure, and the requirement for specific processing (surface pretreatment, curing, process control) [25], the adhesive bonding technique offers several advantages, including a simple process, lower cost, short time, and no need for heating when compared with the above two technologies [26,27,28].

Nelson et al. (1999) fabricated thermal inkjet printheads from two aligned and bonded substrates which were fastened together by a thermosetting adhesive and a UV curable adhesive [29]. However, the thermosetting layer cured at elevated temperatures, resulting in the thermal deformation of the photoresist chamber film structure. Differentiated with respect to previous technologies, the bonding process used in our work only used an ultraviolet (UV) curable polymer as the intermediate adhesive layer, which avoids the thermal deformation of the film structure of the photoresist chamber, as no heating is required. 

Meanwhile, fewer intermediate layer materials are needed, simplifying the process steps. The development of low-cost inkjet printheads is one of the keys to future inkjet printing [30]. Therefore, fabricating thermal bubble printhead chips with practical application value using methods with simpler processes and lower costs is valuable.

In this work, a new process for preparing printhead chips by bonding tantalum nitride (TaN) thin-film heaters and SU-8 chamber film using UV-curable optical adhesive is proposed. The process offers several advantages, including the simplicity of the fabrication step, the ability to fabricate multilayer structures in one photolithography step, and the ability to reuse molds. This process simplifies the preparation process of printhead chips and overcomes the limitations of the traditional adhesive bonding process. Firstly, a chamber film was prepared by the molding lithography process based on PDMS mold. The chamber film was then bonded with the membrane heater by the adhesive bonding process based on film transfer to form a thermal bubble printhead chip. Finally, the chip was integrated with other components to form a thermal inkjet printhead. The results show that the overflow width of bonding interface of 3.10 μm and bonding strength of 3.3 MPa were achieved when the UV curing optical adhesive was spin coated at 6000 rpm and pre-exposed at 320 mJ/cm^2^. Nozzle holes 40 µm in diameter and 338 µm in pitch are suitable for a 75 dpi inkjet print head. In addition, the printhead could stably eject polyvinyl pyrrolidone binder droplets, which are expected to be used for 3D printing of metal or ceramic. These results might provide new clues to better understand the adhesive bonding process based on film transfer and the new applications of thermal bubble printheads. 

## 2. Material and Methods

### 2.1. Design of Printhead Chip Structure

**Figure 1:**
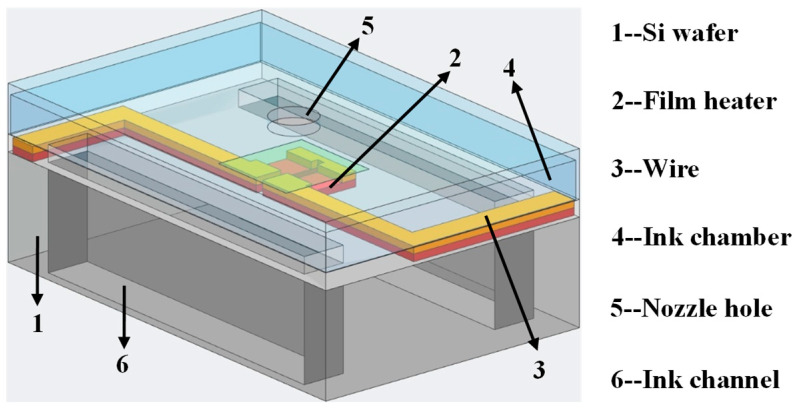
3D model of the inkjet printhead with a single nozzle.

Figure 1 presents the structure of the thermal inkjet printhead with a single nozzle fabricated by the MEMS technology. The size of the ink chamber is 160 × 70 × 15 µm^3^, the nozzle diameter is 40 µm, nozzle pitch is 338 µm, and nozzle number is 16. In this work, the chips of the inkjet printheads were fabricated by the molding method and adhesive bonding method.

### 2.2. Materials 

Commercial negative photoresist SU-8 (MicroChem Corporation, Newton, MA, USA) was used to fabricate the chamber film. Propylene glycol monomethyl ether acetate (PGMEA, Jiangyin Jianhua Microelectronic Materials Co., Ltd., Wuxi, China) was used as a developer to develop the photoresist SU-8. Polydimethylsiloxane (PDMS, Dow Corning, TX, USA) was used as the mold to fabricate the SU-8 chamber film. Silicone film (KRN 500, Hangzhou Guinie New Material Co., Ltd., Hangzhou, China) was used as the glue to bond the transparent glass and SU-8 chamber film. UV curing optical adhesive (NOA 81, Norland, FL, USA) was used as the adhesive to bond the TaN thin-film heater and SU-8 chamber film. The single-side polished 2-inch Si wafer, photomask, and transparent glass are products from the Suzhou Yancai Micro-Nano Technology Co. Ltd. (Suzhou, China). Pigment ink (HP978BK-34, Suzhou RealFast print technology Co., Ltd., Suzhou, China) was used as printing ink to print patterns on a paper substrate. The following materials are products from the Sinopharm Chemical Reagent Co., Ltd. (Shanghai, China): acetone, isopropanol (IPA).

### 2.3. Design and Fabrication of MEMS Process for Printhead Chip Structure

**Figure 2:**
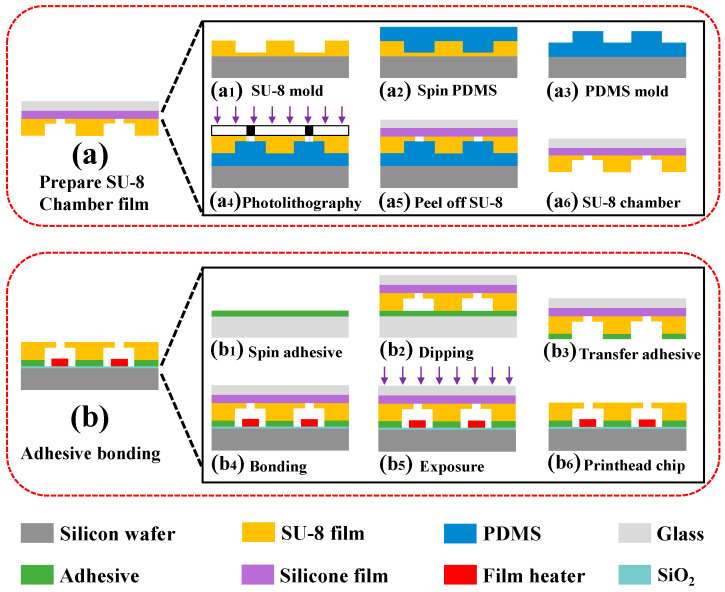
Fabrication schematic of chips for thermal inkjet printheads: (**a**) preparation of SU-8 chamber thin film; (**b**) preparation of printhead chip by adhesive bonding.

Figure 2 schematically shows that the fabrication of inkjet printhead chips mainly includes two process steps. One step is the preparation of SU-8 chamber thin film by molding lithography process using PDMS mold. Another step is the preparation of printhead chip by adhesive-bonding-based film transfer. 

#### 2.3.1. Wafer Treatment

Firstly, the wafers were cleaned with acetone, isopropyl alcohol, and deionized (DI) water. The Si wafers were then dried completely. Afterwards, the wafers were transferred to the gluing machine to remove residual organic contaminants.

#### 2.3.2. Preparation of Chamber and Nozzle Structures 

The schematic of the preparation of chamber film was shown in Figure 2a. First, SU-8 2010 photoresist was spin-coated on a new Si wafer at a spin-coating speed of 1600 rpm for 30 s and soft-baked. The SU-8 layer was crosslinked after UV exposure and post-bake. After developing and hard-baking the uncrosslinked SU-8, an approximately 14-µm-thick structure was fabricated (see Figure 2(a1)). We then poured a degrassed mixture of PDMS prepolymer and curing agent at a weight of 10:1 onto the patterned SU-8 mold and baked at room temperature for 48 h (see Figure 2(a2)). Afterwards, we peeled off the PDMS layer from the SU-8 mold and pasted it onto a transparent glass (see Figure 2(a3)). The SU-8 2015 photoresist was then spin-coated on the patterned PDMS layer at a spin-coating speed of 2800 rpm for 30 s and soft-baked. The SU-8 photoresist was exposed to UV light for 14 s at 140 mJ/cm^2^ (below reference value: 150 mJ/cm^2^) to obtain a transparent and well-shaped nozzle structure layer. After developing and hard-baking, the SU-8 layer was completely crosslinked after a second UV exposure at reference value 150 mJ/cm^2^, resulting in sufficient mechanical strength of the SU-8 layer (see Figure 2(a4)). Finally, we separated the SU-8 chamber film from the PDMS layer by a glass with silicone film attached (see Figure 2(a5)), and obtained the SU-8 chamber with transparent spray hole (see Figure 2(a6)). 

#### 2.3.3. Thin-Film Heater Preparation

Firstly, 100-µm-thick TaN thin film was deposited on a Si wafer covered with SiO_2_ film using a magnetron sputtering system (DE500) at a background vacuum degree of 1 × 10^−6^ Torr, DC sputtering power of 300 W, sputtering pressure of 5 mTorr, nitrogen partial pressure of 15%, and sputtering time of 7.5 min. The Au conductor layer was then deposited by a magnetron sputtering system, and the Au and TaN films were etched to obtain the microfluidic heater structure. Next, SiN_x_ film and Ta film were deposited on the TaN film as protective layers. Finally, the liquid channel was obtained by etching from the back of the Si wafer.

#### 2.3.4. Adhesive Bonding

The schematic of the preparation of the printhead chip by adhesive bonding was shown in Figure 2b. The “stamp and stick (SAS)” transfer bonding technique [31] was used to fabricate the printhead chip. First, the UV curing optical adhesive NOA 81 was spin-coated on a new Si wafer at a spin-coating speed of 6000 rpm for 5 s, and was then exposed at 320 mJ/cm^2^ (see Figure 2(b1)). This process was to reduce the fluidity of the adhesive. Then, the prepared SU-8 chamber film was transferred onto the adhesive NOA 81 layer (see Figure 2(b2)). Next, the chamber film was separated from the adhesive transfer wafer (see Figure 2(b3)). Afterwards, the SU-8 chamber film with adhesive was aligned, attached, and exposed to the TaN heater film by mask aligner, where the exposure energy was 1000 mJ/cm^2^ (see Figure 2(b4,b5)). In order to achieve the optimal bonding performance of the glue, the chip with heater and chamber structure was exposed for 5 min and heated at 50 °C for 12 h using an ultraviolet curing lamp. Finally, the silicon film and glass were peeled off to obtain the chip with heater and chamber structure (see Figure 2(b6)).

#### 2.3.5. Chip Packaging

Firstly, the chip was cleaned with acetone and isopropanol, respectively. The chip dried naturally. A dispenser uniformly dispensed glue with a line width of about 400 µm on the design area of the Al alloy substrate. The chip was then attached to the substrate and cured at 150 °C for 30 min. Next, a bonding machine was used to bond the chip pads and printed circuit board (PCB) pads via gold wires. The gold wire area was protected by dispensing glue on its surface. The Al alloy substrate with the printhead chip was integrated with other components (such as the ink manifold, seal rings, and screws) to form a thermal inkjet printhead.

### 2.4. Characterization

The microstructures of the chip chamber were observed by scanning electron microscope (SEM; Quanta FEG 250, Hillsboro, OR, USA). An ultra-depth three-dimensional microscope (Keyence, VHX-600E, Ōsaka, Japan) was used to observe the microstructures of the chip. The thickness of films was measured by a profilometer (Veeco, Dektak XT, New York, NY, USA). The reflectivity of substrates was measured by spectro-photometer (Jasco, V-660, Tokyo, Japan). A tensile pressure testing machine (Handpi, HLD-100, Shenzhen, China) was used to measure the bonding strength between the films. The viscosity of ink was measured using a viscometer (Fungilab, oneLV, Barcelona, Spain). The surface tension of ink was measured using a surface tension meter (Fungrui, QB2Y-1, Shanghai, China). The drop ejection performance parameters of PVP binder ink were measured by the drop-in-flight analysis system (ImageXpert, JetXpert, Nasuha, NH, USA). The drop ejection performance of pigment ink was measured by the drop-in-flight analysis system (Suzhou RealFast, MDGC, Suzhou, China).

## 3. Results and Discussion

### 3.1. Error Analysis of SU-8 Chamber Film Size

**Figure 3:**
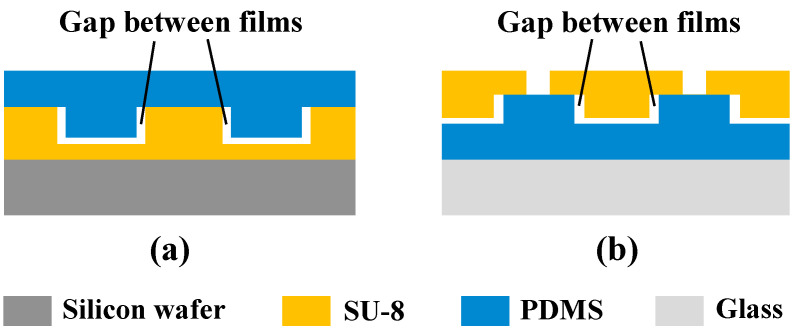
Schematic diagram of shrinkage phenomenon in molding process: (**a**) PDMS shrinkage; (**b**) shrinkage of SU-8 photoresist.

During the two molding processes, the width and depth of the chamber had some errors according to the shrinkage due to SU-8 and PDMS (see Figure 3). The dimension changed during two molding processes, as shown in Table 1. The results show that the width and depth of the chamber of mold #1 were 71.12 µm and 15.31 µm, respectively, and the corresponding error percentages were 1.60% and 2.07%, respectively. For mold #1, such error between mold #1 size and target value is within accepted error determined by the exposure accuracy and measurement precision. In addition, the results show that the width and depth of the chamber of mold #2 were 70.15 µm and 15.11 µm, respectively, and the corresponding error percentages were 0.21% and 0.73%, respectively. Compared to mold #1, mold #2 had a smaller width and depth due to PDMS shrinkage during curing. Additionally, the results show that the width and depth of the chamber of SU-8 chamber film were 70.90 µm and 14.89 µm, respectively, and the corresponding error percentages were 1.29% and 0.73%, respectively. Compared with mold #2, the increase in width and decrease in depth of the SU-8 chamber film was caused by the thermal shrinkage of SU-8 upon cooling or polymerization [32,33].

In summary, the shrinkage of the two materials in the two molding processes resulted in errors in the width and depth dimensions of the SU-8 chamber film, but the error percentages were very small. The error percentage in width between SU-8 chamber film and mold #1 was as low as 0.31% due to the opposite dimensional change direction between SU-8 film and PDMS film in the width dimension. In addition, the error percentage in depth between SU-8 chamber film and mold #1 was approximately 2.80% due to the same dimensional change direction between SU-8 film and PDMS film in the width dimension. Hence, it is necessary to increase the depth of the mold #1 chamber size to compensate when high dimensional accuracy is required. For example, prolonging the time for developing of SU-8 can be used to increase its depth when preparing mold #1.

### 3.2. Characterization of Bonding Strength of Each Interface

**Figure 4:**
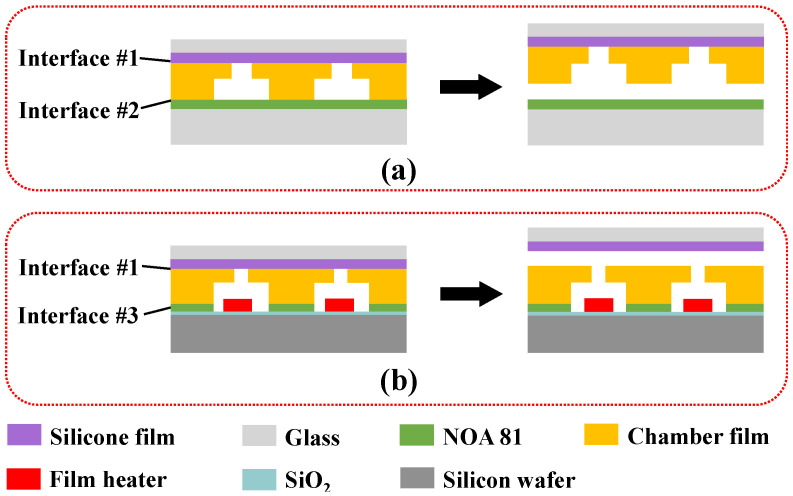
Interfaces involved in film transfer method: (**a**) demolding; (**b**) bonding.

As shown in Figure 4, the SU-8 compartment film was transferred from the PDMS mold to the transfer carrier and then from the transfer carrier to the heater surface after the two molding processes were completed. In order to ensure that the film transfer process could be carried out successfully, the bonding strength of the interface #2 was less than that of the interface #1 during the demolding process, and the bonding strength of the interface #1 was less than that of the interface #3 after bonding. Therefore, a tensile pressure testing machine was used to measure the bonding strength of the interface, and the results are shown in Table 2. The results show that the bonding strength of interface #3 was much greater than that of interface #1 and interface #2, which is conducive to separating the SU-8 chamber film from the silicon film. In addition, it can be seen from the results that the bonding strengths of the three interfaces satisfy the following relationship: interface #1 < interface #2 < interface #3, which meets the requirements of the interface bonding strength during the film transfer process.

The optical image of adhesive bonding interface is shown in Figure S1. It can be observed that there were several small bubble defects around the chamber after the bonding process was completed. The air bubbles are squeezed away from the chamber boundaries due to the concentration of bonding pressure at the chamber boundaries. Although the appearance of bubbles reduced the bonding strength, the bonding strength still met the requirements. Further studies are needed to reduce the air bubbles by improving bonding process.

### 3.3. Control Analysis of the Mount of Adhesive Overflow

**Figure 5:**
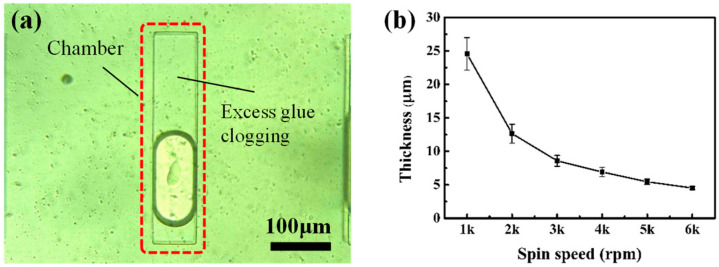
(**a**) Chamber clogging caused by adhesive overflow; (**b**) the spin speed–thickness curve of adhesive NOA 81.

The UV-curing optical adhesive NOA 81 is a one-component glue with a photosensitive wavelength range of 320–380 nm and a peak sensitivity at 365 nm. The adhesive layer after curing still has a certain degree of elasticity, which can prevent the vibration of the inkjet printhead chip during operation from damaging the bonding interface. Generally, the lower viscosity of adhesive NOA 81 is easy to separate, which is conducive to the dipping and transfer of the glue. However, the excessively low viscosity also makes the glue have strong fluidity. As the interface needs to be contacted through pressure during the dipping and bonding process, the pressure will make NOA 81 glue flow into the chamber structure, resulting in chamber blockage, as shown in Figure 5a. In order to solve the problem of chamber blockage, two methods of high-speed spin-coating and precuring were used to control the amount of adhesive overflow. 

High-speed spin-coating. Using a higher spin speed for the spin coating of the adhesive NOA 81 can result in a thinner adhesive layer and a smaller amount of adhesive overflow, which can reduce the amount of adhesive overflow during the dipping process. In order to obtain the speed–thickness curve of NOA 81, the Si wafer was spin-coated with adhesive NOA 81 at 6000 rpm as a carrier film, and was completely crosslinked after exposure for 10 min by UV curing lamp. Then, acetone was used to erase the adhesive layer at the edge of the wafer to prevent the edge bead phenomenon from causing errors in the thickness measurement. The thickness of the residual adhesive layer was measured by a profilometer, and the result is shown in Figure 5b. The results show that the thickness of the glue layer decreases with the increase of the rotation speed. When the rotation speed was 6000 rpm, the thickness of the glue layer was reduced to 4.53 µm, which is less than 1/3 of the depth of the chamber film. Although continuing to increase the spin speed can make the thickness of the adhesive layer lower, excessive spin speed may damage the spin-coater and shorten its service life. Therefore, 6000 rpm was finally selected as the spin coating speed of the UV curing optical adhesive NOA 81.

**Figure 6:**
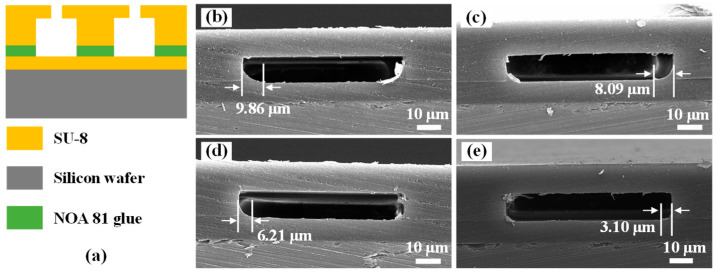
(**a**) The schematic diagram of cross-sections of test samples; SEM images of cross-sections of test samples exposed at various exposure doses: (**b**) 260 mJ/cm^2^; (**c**) 280 mJ/cm^2^; (**d**) 300 mJ/cm^2^; and (**e**) 320 mJ/cm^2^.

Precuring. Precuring was chosen to reduce the fluidity of adhesive NOA 81, thus reducing the amount of adhesive overflow. For the convenience of expression, the degree of NOA 81 curing is expressed by the dose of pre-exposure. The Si wafer was spin-coated with adhesive NOA 81 at 6000 rpm as a carrier film and exposed to various exposure doses. Then, the adhesive overflow test samples were prepared according to the adhesive dipping and bonding process. The width of the adhesive overflow was measured by observing the cross section of the sample after the second exposure and aging treatment by SEM; the results are shown in Figure 6. The step height on the surface of the fully exposed remaining adhesive layer (i.e., the amount of adhesive transfer) was measured by a profilometer, and the results are shown in Table 3. 

It can be seen from Table 3 that the adhesive transfer amount and adhesive overflow width decreased with the increase of the pre-exposure dose when the pre-exposure dose was in the range of 260–320 mJ/cm^2^. The influence of pre-exposure on the width of the adhesive overflow can be clearly seen in Figure 6. These results demonstrate that the precuring treatment can significantly improve the problem of adhesive overflow in the bonding process. However, when the pre-exposure dose exceeded 320 mJ/cm^2^, due to the high curing degree and high viscosity of adhesive NOA 81, the SU-8 chamber film was adhered to the adhesive film and desorbed with the silicone film during the dipping process, leading to the failure of adhesive transfer.

### 3.4. Analysis of the Effect of Adhesive Bonding

**Figure 7:**
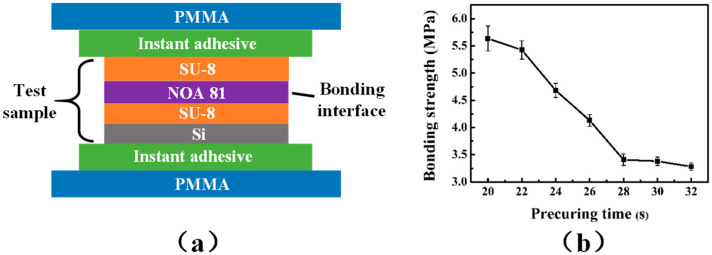
(**a**) Schematic diagram of the sample used for bonding strength test; (**b**) the precuring time-strength curve.

In order to ensure that the bonding interface of the inkjet printhead chip will not be separated or damaged due to the impact of thermal bubbles during operation, the bonding strength is required to be greater than 0.9 MPa [34]. The bonding strength produced by adhesive NOA 81 at different pre-exposure doses was tested using a tensile pressure testing machine. The structure of the test samples is shown in Figure 7a, and the sample size was 10 mm × 10 mm. The PMMA plastic at both ends of the test sample was fixed in the upper and lower slots of the testing machine. Stress values at fracture were recorded as the samples were stretched to fracture. Three specimens per experimental point were measured to ensure the reliability of the data. The results of bond strength are shown in Figure 7b. It can be seen that the bonding strength decreases with the increase of pre-exposure dose, i.e., an increase in pre-exposure dosage will have an adverse effect on bonding strength. Therefore, high bonding strength can be obtained by reducing the pre-exposure dose. The experimental results show that the minimum bond strength is 3.3 MPa, which is much greater than 0.9 MPa. Hence, the bonding strength generated by adhesive NOA 81 exposed at different pre-exposure doses always met the bonding property requirements.

### 3.5. Analysis of Droplet Flight of the Printhead

**Figure 8:**
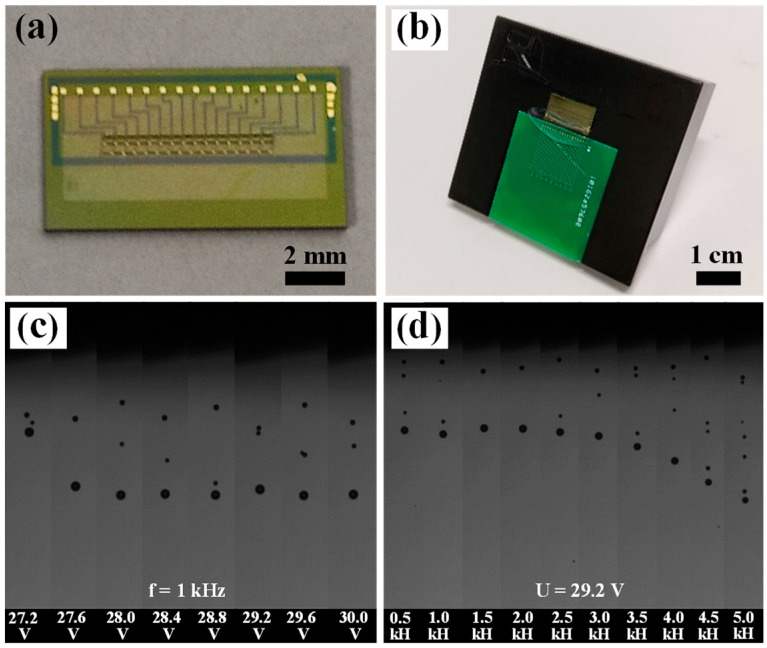
The optical image of (**a**) the chip and (**b**) the printhead; series of driving voltage (**c**) and jet frequency (**d**) recorded by droplet flight analysis system.

In order to verify whether the bonding interface can withstand the impact of thermal bubbles and whether the printhead can eject stable ink droplets, we observed the flying state of the ink using an ink drop observer. The test ink was a polyvinyl pyrrolidone (PVP) binder ink with a density of 1.05 g/cm^3^, a surface tension of 29.6 mN/m, and a viscosity of 2.99 mPa·s. The type of signal wave used in the test was a rectangular wave with a pulse width of 1 μs (see Figure S2). Figure 8a,b show the image of the chip and the packaged thermal bubble printhead, respectively. Meanwhile, the optical image of the nozzle hole with a diameter of 40 μm is shown in Figure S3. The images of droplet flight under a driving voltage and jet frequency series are shown in Figure 8c,d. As shown in Figure 8c (the jet frequency was set as 1 kHz), the main droplet was formed with a few satellite droplets when voltage increased to 27.2 V. The main droplet velocity was low, which was caused by the insufficient energy of the TaN thin film resistor at low voltage. As the driving voltage further increased, the main droplet velocity increased and then became stable. The change of droplet velocity with driving voltage is consistent with the standard curve of thermal bubble inkjet [35]. As shown in Figure 8d (the driving voltage was set as 29.2 V), no obvious change in droplet volume or velocity can be observed when jet frequency was in the range of 0.5–3.0 kHz. When jet frequency increased from 3.0 kHz to 5 kHz, the droplet velocity volume continued to increase and droplet volume began to decrease. 

In addition, the reliability of the printhead was analyzed. The results show that the nozzle can continuously eject ink for 12 h at a voltage of 29.2 V and a frequency of 1 kHz. After the test, the printhead could still stably eject ink when used again. This proves the bonding interface can withstand the impact generated by the inkjet printhead without being damaged. Therefore, it demonstrates that the bonding strength can meet inkjet requirements.

However, the formation of satellites is typically an unwanted feature of inkjet printing technology [36,37]. Liu et al. reported that the threshold for satellite formation is a function of *Z* and the practical range for ink printability is approximately bound by 2 < *Z* < 20 [38]. $Z=\sqrt{\gamma \rho a}/\eta$, where *γ*, *ρ*, *η* and a are the surface tension, density, dynamic viscosity of the fluid, and nozzle diameter, respectively [39]. Meanwhile, they reported the low-viscosity fluid formed liquid columns that readily pinched off to form satellites [28]. As shown in Table 4, *Z* is 11.7 for PVP binder ink, which meets the practical range for ink printability reported by Liu et al. [38].

In order to visually observe the printhead performance, pigment ink was ejected to the paper substrate by the printhead. The black pigment ink consisted of water, coloring agent, alcohol solvent, and surface-active agent. Figure S4a gives sequences of photographs of the drop formation at different delay times for pigment ink (inset image is the optical image of black pigment ink). The driving voltage was 30 V. The main droplet and satellite droplet are shown. Figure S4b shows the printed droplet patterns at different jetting frequencies. It can be seen that the pattern became darker as the ejection frequency increased, indicating an increase in the number of ink droplets. In addition, there are no black imprints around the pattern, indicating that the main droplets and satellite droplets can be ejected into the printing area. The print pattern has a low definition, which is caused by the lower resolution of the printhead (75 dpi). The printhead can be designed with a higher resolution in future research to improve the resolution of the printed pattern. 

Huang et al. used a thermal bubble printhead fabricated using a sacrificial layer process to eject a low viscosity of water-based GO ink [8]. In their work, the 0.8 mg/mL GO ink has a density of 0.89 g/cm^3^, surface tension of 23.1 mN/m, viscosity of 3.28 mPa·s, and *Z* value of 7.57. In their drop-in-flight experiment, the GO ink was stably ejected, but satellite droplets could clearly be observed. In our work, the thermal bubble printhead fabricated using an adhesive bonding technique was also used to eject water-based PVP ink. The PVP ink had a density of 1.08 g/cm^3^, surface tension of 29.6 mN/m, viscosity of 3.05 mPa·s, and *Z* value of 11.7. Compared with Huang’s work, the printhead fabricated by adhesive bonding technique can also be used to eject low viscosity ink. In addition, the method used in our work has a simpler process and lower fabrication cost.

## 4. Conclusions

In this article, a new process for preparing printhead chips by bonding TaN thin film heaters and SU-8 chamber film using UV-curable optical adhesive was proposed. The process offers several advantages, including the simplicity of the fabrication step, the ability to fabricate multilayer structures in one photolithography step, and the ability to reuse molds. This process simplifies the preparation process of printhead chips and overcomes the limitations of the traditional adhesive bonding process. Firstly, a chamber film was prepared by the molding lithography process based on a PDMS mold, and then the chamber film was bonded with the membrane heater by the adhesive bonding process based on film transfer to form a printhead chip. Finally, the chip was integrated with other components to form a thermal inkjet printhead. The results show that a printhead chip with a complete structure and minimal adhesive overflow was fabricated. The bonding strength of 3.3 MPa was achieved when the exposure dose was set at 320 mJ/cm^2^, which meets the bonding strength requirement. In addition, the printhead could stably eject PVP binder droplets, which are expected to be used for binder-jetting printing of powder such as ceramics, metals, and sand molds. These results might provide new clues to better understand the adhesive bonding process based on film transfer and the new applications of the thermal bubble printhead. Additionally, they are valuable for future research on the uniformity of ink deposition in inkjet printing of this printhead. Although the adhesive bonding approach has some disadvantages, such as the need to have adherent substrates and the requirement for specific processing (surface pretreatment, curing, process control), we believe that this approach is expected to be used for the small batch industrial production of printheads via aligning and bonding of films and wafers on lithography machines at room temperature.

## Figures and Tables

**Table 1:** Dimension changes during molding process.

	Width (µm)	Width Error (%)	Depth (µm)	Depth Error (%)
Target value	70	0	15	0
Mold #1	71.12	1.6	15.31	2.07
Mold #2	70.15	0.21	15.11	0.73
SU-8 chamber film	70.9	1.29	14.89	−0.73

**Table 2:** The bonding strength of each interface.

Interface	Material	Bonding Strength (MPa)
#1	SU-8/Silicon film	0.95
#2	SU-8/PDMS	0.46
#3	NOA 81	27.58

**Table 3:** The adhesive transfer amount and adhesive overflow width at different exposure doses.

Exposure Doses (mJ/cm^2^)	Adhesive Transfer Amount (µm)	Adhesive Overflow Width (µm)
260	1.61	10.29
280	1.31	8.09
300	1.25	6.21
320	1.08	3.10

**Table 4:** Composition and fluid physical properties of the PVP binder ink, together with the computed value of the dimensionless number *Z*, taking the characteristic length to be the diameter of the printing orifice (40 µm).

Composition	Density (g·cm^−3^)	Dynamic Viscosity (mPa·s)	Surface Tension (mN·m^−1^)	*Z*
PVP, DI water, DEG, Dynol-465	1.08	3.05	29.6	11.7

## Supplementary Materials

The following supporting information can be downloaded at: https://www.mdpi.com/article/10.3390/mi13030455/s1, Figure S1: The optical image of adhesive bonding interface; Figure S2: Schematic of the rectangular waveform used with RF-T16 droplet generator; Figure S3: The optical image of nozzle hole with the diameter of 40 μm; Figure S4: (a) Time series droplets recorded with a drop-in-flight analysis system for pigment ink (inset image is the black pigment ink); (b) the optical image of printed droplet patterns at different jetting frequencies.
