# Supplementary material for: A Simple Method for Fabricating Ink Chamber of Inkjet Printheads
Source: Micromachines (Basel). 2022 Mar 17;13(3):455. doi: 10.3390/mi13030455 (PMC8949317; doi:10.3390/mi13030455)
Supplement: Supplementary file 1 [file micromachines-13-00455-s001.zip › micromachines-1581334-supplementary.pdf]

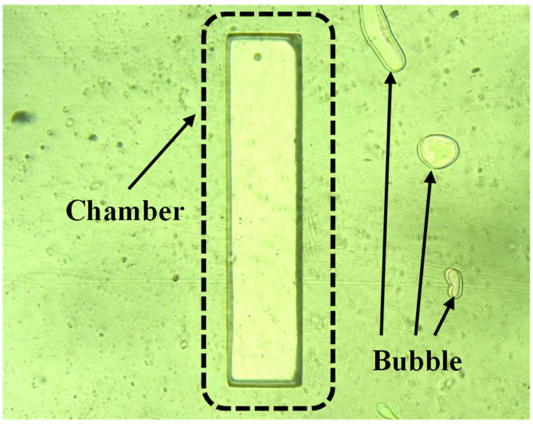

Figure S1. The optical image of adhesive bonding interface.

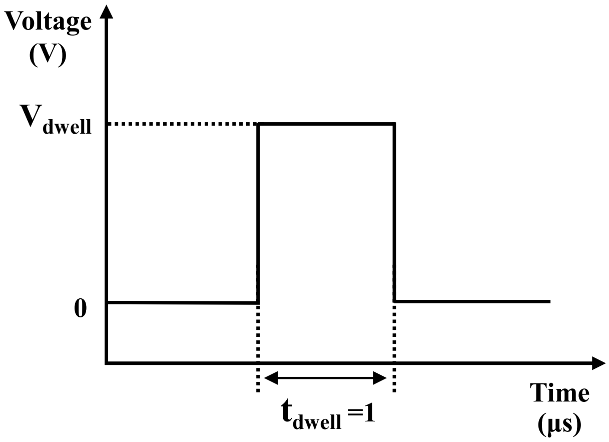

Figure S2. Schematic of the rectangular waveform used with RF-T16 droplet generator.

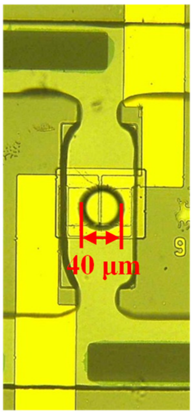

Figure S3. The optical image of nozzle hole with the diameter of 40 μm.

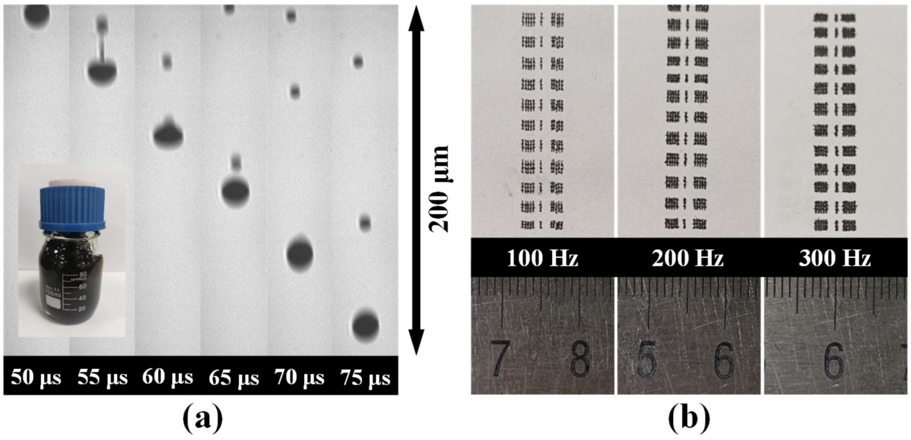

Figure S4. (a) Time series droplets recorded with a drop-in-flight analysis system for pigment ink (inset image is the black pigment ink); (b) the optical image of printed droplet patterns at different jetting frequencies.
